# Experiences of family caregivers of patients with COVID-19

**DOI:** 10.1186/s12875-021-01489-7

**Published:** 2021-06-29

**Authors:** Tahereh Rahimi, Neda Dastyar, Foozieh Rafati

**Affiliations:** 1 Department of Public Health, School of Health, Jiroft University of Medical Sciences, Jiroft, Iran; 2 Department of Midwifery, School of Nursing and Midwifery, Jiroft University of Medical Sciences, Jiroft, Iran; 3 Department of Nursing, School of Nursing and Midwifery, Jiroft University of Medical Sciences, Jiroft, Iran

**Keywords:** Family caregivers, COVID-19, Experiences, Phenomenology

## Abstract

**Background:**

Family caregivers of patients with COVID-19 face many challenges that affect their physical and mental health.

**Aim:**

The aim of the present study was to explore experiences of family caregivers of patients with COVID-19.

**Methods:**

This phenomenological study was performed based on 13 family caregivers who had experience in home caring for patients with COVID-19. Data were collected through purposive sampling with in-depth semi-structured interviews. The Colaizzi's 7-step method was used to determine themes. The MAXQDA10 software was used to manage qualitative data analysis.

**Results:**

Thirteen family caregivers participated. Five main themes describe family caregivers' experiences of caring for patients with COVID-19: nature of the disease; unmet needs; unpleasant physical, psychological, and social experiences; care facilitators and positive experiences.

**Conclusion:**

Information and financial support for COVID-19 should be provided to family caregivers. Also, community members should embrace patients and family caregivers and reinforce the positive experiences of caregivers.

**Supplementary Information:**

The online version contains supplementary material available at 10.1186/s12875-021-01489-7.

## Background

The coronavirus disease 2019 (COVID-19) is a worldwide epidemic infection, which first reported in Wuhan, China, in December 2019, and it rapidly became a growing public health concern with high transmission probability [1]. Based on the latest WHO statistics on May, 2020, more than 160 million cases of COVID-19 had been confirmed, which resulted in over 3 million deaths globally. The number of patients in the United States, India and Brazil is higher than other countries and Iran is ranked the fourteenth in the world [2].

Although increased risk of severe illness and death has been more observed in the older adults and individuals with underlying medical conditions, current research evidences show that all ages are susceptible to coronavirus infection [3, 4].

Since there is no specific cure for COVID-19, treatment is limited to symptomatic and supportive therapy [5]. Patients with severe COVID-19 need to be hospitalized and mild or some moderate patients without underlying chronic conditions can receive care from family caregivers [6]. The family caregivers are usually family members, or close relatives who provide care, typically voluntarily to patients at home [7]. They provide significant support to individuals who are elder or have chronic disease, disabilities, mental health problems, and addiction. The growing prevalence of COVID-19 and the lack of hospital facilities and space needed to care has caused the early discharge of COVID-19 patients from the hospital. Therefore, these patients have become also a new target group for receiving home care. The family caregivers are reducing increased pressure on health and social care systems [6, 8]. Assistance with performing activities of daily life, managing treatment-related conditions, communicating, educating, encouraging and empowering the patients to take care of themselves is known as responsibilities of home caregivers [8, 9].

A collection of positive and negative experiences may be experienced when family caregivers move into their caregiving role. They often expose to physical and psychological needs, such as caregiving overload and emotional stress [10]. Several factors are associated with caregiver distress, including incarceration, lack of leisure time, lack of assistance from other family members, poor care knowledge, caregiver age, and guilt over ignoring the patient's complaints [11–13]. Vagueness, stigma, discrimination, change in relationships with the patient and others, and compassion fatigue may also be experienced as negative experiences [14, 15]. Feeling good about themselves, compassion satisfaction, learning new skills, and strengthening family relationships are some of the positive experiences mentioned by family caregivers [15, 16]. Findings of scientific researches show that different experiences of family caregivers may be associated with their physical and mental health [10, 11, 16].

In general, the characteristics of caregiver burden are common in physical and mental diseases. Family caregivers often affected by the biological and physiological damage caused by patient care process and also would probably face reduced job, social activities and relationships with family and friends. However, this should not be ignored that different disease have specific effects on caregivers due to differences in symptoms, treatment and social reaction to them and make special needs for care of patients [16, 17]. The different nature of COVID-19 and the need to maintain public safety, will limit the physical presence with patients, and therefore caregiving in this situation will be unique for everyone involved. The importance of maintaining social distance to control the COVID-19 pandemic probably increased isolation, loneliness, psychological stress and other adverse health problems [18]. Social and family relationships are disrupted not only for patients but also for their caregivers [19]. The caregivers of patients with COVID-19 face greater challenges compared to other caregivers because of limited training and resources at hand and their lack of knowledge about this emergent disease and the way to care for the patient [20].

In Iran, especially in smaller cities and rural areas such as Jiroft, the community members are not indifferent to the family and their neighbors. Given the religious and cultural values and strong family relationship, Iranian family caregivers who are often women, actually are willing to take care of the patients and provide a strong support network to them. But the COVID-19 conditions have overwhelmed family caregivers, as well as others in the community, with new economic stressors such as job loss by the household head and high medical expenses [21]. They have to take care of their patients all by themselves. Many family caregivers have other roles in addition to the caregiving role such as managing their job, housekeeping, child care and additional responsibilities and concerns about their children's education due to school closures from COVID-19. They also must strive to protect themselves and other family members from virus transmission and all of this is more difficult to manage than caring for other disease or in other situations.

However, to date, no study has been conducted on family caregiving experiences of caring for patients with COVID-19. Most of studies have focused on the experiences of patients or health care staff. For example, death anxiety and fear, depression, stigma, ambiguity and decreased communication with family and society reported as COVID-19 patients' experiences [22]. Moreover, mental and emotional distress and working in inadequate professional conditions were experienced by nurses who cared for the patients with COVID-19 [23]. While the evidence shows home-based caregiving effects on caregiver’s quality of life and life satisfaction and so the consideration of caregivers' experience is necessary to plan services and support them [24]. The present study was conducted to understand the experiences of family caregivers for patients with COVID-19 to explore their challenges regarding this novel disease.

## Materials

### Study design

A descriptive phenomenological methodology was adopted to explore the family caregivers' experiences of caring for patients with COVID-19. The phenomenology is an approach that enables the researcher to explore the lived experiences of individuals as they occurred in life [25]. There is evidence to show that phenomenology have been used as an effective research method to understand people's experiences in health and illness and perceived caring needs of patients and those who take care of them [26]. Two main phenomenological approaches identified as interpretive and descriptive. Contrary to the interpretive phenomenological approach that focuses on interpreting and exploring hidden meanings of the phenomenon described, descriptive phenomenological approach is applied when the researchers have little information about issue such as care experiences related to a newly emerging disease (COVID-19). In this approach, the purpose of conducting research is to clarify and understand the most essential meaning of a phenomenon under study from the perspective of people who are directly involved in it [27]. For example in the descriptive phenomenology study, the researcher may ask the general question such as, “Tell me what it is like to be a working mother” and follow up with questions to reach common concepts related to the experience. On the other hand, interpretive phenomenologist, would be sure to the working mother’s description of a typical day in detail, and would encourage them to more describe daily workload, interpersonal relationship, body's experiences and other hidden lived experiences occur in the context of daily work practices and socialization.

The openness to the phenomenon under study must be emphasized when researchers investigate lived experiences. The openness emerged when researchers had questioned their preunderstanding, which means recognizing and becoming informed of preconceptions that might affect the analysis. This is the same as bracketing, a generally used term in descriptive phenomenology [28]. The bracketing means to waive the researcher's prior knowledge about the study phenomenon. Bracketing helps to better understand the essence of the phenomenon from the participants' viewpoint [29]. In this study bracketing was considered in every part of study process, especially in analysis. In order to do this, the authors used a reflexive diary to write their previous feelings and perceptions and also recorded field notes. The interviews were read over and over again to ensure that the real experiences of the participants reflected without any bias. In addition, some family caregivers were asked to comment on the content of the analyzed interview to clarify their perceptions and experiences.

### Sampling and participants

The study was conducted in 2020 in Jiroft, which is located in the southeast of Iran. The inclusion criteria included caregivers who were responsible for taking care of patients with COVID-19 at home, and who had the ability to communicate properly. Purposive sampling was used to identify participants for the study. This sampling method applies to generate a homogenous sample of participants that have all experienced the study phenomenon [30]. Additionally, maximum variation in terms of age, gender, educational levels, occupational status and residential location was considered to obtain comprehensive and rich data. Sampling was continued until saturation of themes was achieved.

### Data collection

Data were collected from Jun to Aug 2020. First, a list of telephone number of patients diagnosed with COVID-19 disease was prepared from the city health center and then the contact information of home caregivers was obtained. The objectives of the study, the method of data collection, and the voluntary participation in the study were explained to participants. The time and place of the interviews were determined by the caregivers. All but one (at caregiver home) interview was conducted in the hospital. In-depth semi-structured face to face interviews was conducted to encourage the participants to discuss their experiences from caring for a patient with COVID-19.

The open-ended interview guide was designed and validated by the research team and included questions such as “Please describe your caring experience of patient with coronavirus disease in as much detail as possible." When necessary, probes and follow-up questions (e.g., can you please explain more? can you give an example?) were added to encourage discussion and clarify answers. Every interview session ended with the question “Is there anything else you want to add, which I did not ask?” Interviews lasted 30 to 70 min, were digitally audio-recorded with participants’ permission, and transcribed verbatim. To ensure data confidentiality, an ID number was assigned to each participant and the transcripts were without personal information.

Data were collected until data saturation was reached. After interviewing 11 participants, it was observed that no additional data could be obtained. To ensure that there was no other new information; the researchers conducted 2 more interviews.

### Data analysis

All interviews were recorded and transcribed verbatim by ND. In this study, data were analyzed using Colaizzi's 7-step method of data analysis [31]. Firstly, two of the authors (FR, TR) reviewed the transcribed interviews individually several times to achieve a general sense about whole content obtained. In the second step which is known as, significant statements and phrases related to caring experience of family caregivers for patient with COVID-19 were extracted. In the third step, the authors (FR, TR) determined and formulated meanings from these significant statements and then the meanings of statements were organized into categories, clusters of themes, and themes in the next step. To create a comprehensive description of the experience described by family caregivers; in the fifth step, the authors compared their findings to the transcripts and confirmed that emerging themes described the essence of the participant's statements. Ambiguities and disagreements were discussed by the authors until a consensus was reached on the analysis. Then, fundamental structure of the phenomenon was described. The authors constantly tried to disregard their beliefs and opinions throughout analytical steps. Finally member checking was used for validation of the findings with family caregivers. For this purpose, ND returned the research findings to the caregivers and discussed the results with them. Participants' comments on the study results were obtained by telephone and they confirmed that the results are consistent with their experiences. The MAXQDA 10 software (VERBI GmbH, Germany, 2010) was used to manage and organize the qualitative data. Examples of analysis process are shown in Table 1.

**Table 1:** Examples of the analysis process

Meaning units	Condensed Meaning units	Subthemes	Themes
My husband was visited only once. The doctor told him to go home and not go out for 14 days. He gave him only two pills and said that because him general condition was not very bad and he had no underlying disease, he did not need to go to the hospital. He said if he gets very ill, take him to the emergency room, that's all! But this was not enough at all for the thousands of questions that were created in my mind, and I wish these patients and their families were not left like this, saying these few sentences is not enough at all	Lack of doctor's enough explanation about the disease and patient care	Lack of knowledge	Unmet needs
It would be great if there was a situation where it was possible to make a video call to the doctor to visit the patient remotely. but unfortunately this was not possible	lack of telemedicine	Lack of health facilities	

### Trustworthiness

Demonstrating trustworthiness is essential for qualitative studies, which are measured by criteria proposed by Lincoln and Guba, including credibility, transferability, dependability, and confirmability [32]. In this study, prolonged engagement (through communicating with the family caregivers for about two months of direct involvement and one month for the member check and also providing multiple interviews on average 50 min), member checking (through giving some transcribed interviews to family caregivers and asking them whether the description is complete and realistic, according their experiences.), peer debriefing, thick contextual description, and enabling external audits were implemented as trustworthiness strategies.

### Ethics

The study design and guideline interview was approved by the Scientific and Ethical Committee of Jiroft University of Medical Sciences (Ethics number IR.JMU.REC.1399.019) and all methods were performed in accordance with the relevant guidelines and regulations. Written informed consent was obtained from all participants. To ensure anonymity and confidentiality, identifying information of family caregivers was not transcribed and all participants were given fictitious names and descriptions.

## Results

The age of the participants ranged from 22 to 54 years. Twelve of the participants were female and 1 was male. Nine of the participants were married. All caregivers cared for their first-degree relatives (mother, father, child, sister, and spouse) at home. After analyzing data, 335 initial concept codes, 14 sub-themes and 5 main themes were emerged including (a) Nature of the disease; (b) Unmet needs; (c) Unpleasant physical, psychological, and social experiences; (d) Care facilitators; and (e) Positive experiences.

### Nature of the disease

This theme referred to the caregivers' experience about caring for a patient with the COVID-19. They stated that the experience of caring for a patient with COVID-19 was different from their previous caring experiences. This theme included 2 subthemes: “fluctuating symptoms” and “emergent and unpredictable disease.”

#### Fluctuating symptoms

This subtheme indicated the caregivers' experience of fluctuating symptoms of the disease. According to the caregivers, recurrence of the disease symptoms after partial relief, reversal of the symptoms, and the appearance of unexpected symptoms caused them to feel hopelessness, fear, and stress. They stated that as their patients' symptoms fluctuated, they also fluctuated between hopelessness and hopefulness, fear and reassurance, and stress and comfort. In this regard, one of the caregivers said:*“My husband’s fever stopped for a few hours, but I did not know whether to help or not because the fever started again and this happened over and over again. Sometimes, he was feeling good and sometimes he was feeling bad; such fluctuating symptoms bothered me.”* (Caregiver 5, 34 years old).

#### Emergent and unpredictable disease

Most caregivers felt anxiety in dealing with this disease because COVID-19, as an emerging and unknown disease, has no definitive treatment and its course is unpredictable. Ambiguity and uncertainty about the unknown consequences and prognosis of the disease also increased caregivers' anxiety.

“This is an emergent disease; even physicians do not know much about it. It is evident that caring for these patients is difficult.” (Caregiver 1, caring for a sister, 22 years old).

“It was a really difficult experience given that the disease is unknown; I did not know how the disease would progress.” (Caregiver 4, caring for a husband, 37 years old).

### Unmet needs

This theme was about the needs of caregivers that were not met and made the care experience challenging and difficult for caregivers. The subthemes were *lack of knowledge*, *lack of health facilities*, and *financial problems*.

#### Lack of knowledge

All participants considered lack of knowledge and care skills as one of the main problems in caring for patients with COVID-19. Most caregivers expressed that they doubted their ability to care, their information needs were not met, and they feared that this lack of knowledge and skills would cause harm to the patient. Caregivers also stated that they applied all of their previous care knowledge and experience, but the patient's symptoms did not improve significantly or recur. They felt were abandoned and helpless. One of the caregivers described her experience as follows:

“When my mother's shortness of breath got worse, I did not know what I was doing was right or not. I told myself ‘I may endanger her life and make her worse.’ It would have been great if I had a source to guide me.” (Caregiver 2, caring for a mother, 41 years old).

#### Lack of health facilities

According to the caregivers, problems caused by lack of health facilities included lack of access to health care services, lack of access to physicians, lack of telemedicine, and lack of counseling center and psychological support. They needed health care to be available and accessible 24 h a day. They complained about they had to take the patient out of quarantine and even wait a few hours for the doctor to visit their patients in some cases. Also, they needed counseling services to reduce their own and the patient's psychological symptoms.

One of the caregivers said, *“If there was a special care center for COVID-19 patients that provided 24-h services, people did not have to spend a lot of time visiting physicians. Consequently, stress and anxiety would have been reduced in caring for the patients.”* (Caregiver 2, caring for a mother, 41 years old).

“Considering the quarantine situation, we do not want to leave the house. I wish some physicians visited the patients using WhatsApp or video calls.”(Caregiver 9, caring for siblings, 27 years old).

#### Financial problems

This subtheme was extracted from the statements of the caregivers who cared for male patients with nongovernmental jobs. These participants indicated that they had financial needs during the quarantine and mentioned that the government should support patients with financial and health packages. They stated that on the one hand, their income decreased and on the other hand, treatment expenses were added to their other expenses during the COVID-19 outbreak.

“My husband is self-employed; he could not leave the house for almost 3 weeks due to the disease and we were in financial difficulties. The government should provide financial assistance to financially weak families.” (Caregiver 5, caring for a husband, 34 years old).

Quarantine, loneliness, illness, and financial problems affected our mental health. The government must help us (Caregiver 6, caring for a mother, 26 years old).

### Unpleasant physical, psychological, and social experiences

Caregivers described experiences that were physically, psychologically, or socially unpleasant and even painful at time. This theme consisted of the following subthemes: unpleasant physical experiences, unpleasant psychological experiences, and unpleasant social experiences.

#### Unpleasant physical experiences

Some caregivers reported experiencing physical problems, such as sleep disturbances, anorexia, and allergies caused by overuse of disinfectants, and fatigue. Fatigue and insomnia were the most commonly reported physical symptoms *“I did not sleep until the wee hours of the morning because I was worried and anxious about my husband. I wanted to pay all my attention to him.”* (Caregiver 13, caring for a husband, 54 years old).

#### Unpleasant psychological experiences

All participants had unpleasant psychological experiences. They described this period as difficult and terrifying. These experiences were varied, but the most common ones were fear, anxiety, worry, sadness, hopelessness, and mental preoccupation about the disease outcome, and feeling powerless to manage the patient^'^s symptoms. A caregiver mentioned,

“My mind was occupied by thinking about my mother. I thought she may get worse and die, God forbid! All these thoughts made me sad, upset, depressed, and frustrated.” (Caregiver 6, caring for a mother, 26 years old).

“I had a dark and sad period.” (Caregiver 10, caring for a 4-year-old child, 32 years old).

#### Unpleasant social experiences

Caregivers' unpleasant social experiences, including experiences occurring when they encountered negative reactions from healthy people in the community. They felt socially rejected and deprived because their friends and family members avoided contacting them because they thought the caregivers may be disease carriers. Moreover, they were home alone with the patient, which made them feel lonely.

“Even after the quarantine period was over, others would distance themselves from me and shout 'Why did you come out?' and I felt very upset.”(Caregiver 2, caring for a mother, 31 years old).

### Care facilitators

This theme included factors that facilitated the care process for caregivers and included subthemes *of social support*, *adaptive mechanisms*, and *intrinsic motivations*.

#### Social support

According to the caregivers, social supports entailed practical supports, such as providing food and necessities of life and guidance for care as well as emotional support. Relatives and friends showed emotional support for caregivers by offering genuine encouragement, reassurance, empathy, and compassion. Social support encouraged them to continue caring with strength.

“My family members phoned and encouraged me. This reduced the loneliness and severity of the caring process. (Caregiver 3, caring for a husband, 29 years old).

#### Adaptive mechanisms

All caregivers used some mechanisms to reduce care-related stress. They used a combination of problem-oriented and emotion-oriented strategies to reduce their stress. The most common defense mechanisms used by caregivers were: positive self-talking, distracting, seeking information from various sources, as well as praying and trusting God.

“When my mind was occupied with illness and death and fear took me over, I immediately distracted myself by doing something or playing with children.” (Caregiver 7, caring for a husband, 37 years old).

“I got the necessary information about the disease from TV, Telegram, and Instagram” (Caregiver 6, caring for a mother, 26 years old).

I told my family don't send negative energy and I kept saying to myself: I am sure that they (sick parents) are fine and do not need hospitalization. (Caregiver 8, caring for parents, 24 years old).

#### Intrinsic motivation

Participants expressed a sense of responsibility regarding the patient and family health. The interest between caregiver and care recipient as well as observation of the healing process (as an internal driver) made the caregivers enjoy caring for the patient rather than performing a task that is imposed on them. A male caregiver who cared for his wife indicated, *“I accepted the responsibility to take care of my wife to protect our life and children. So, I took care of my wife with all my heart and soul.”* (Caregiver 12, caring for a wife, 48 years old).

### Positive experiences

Based on the participants' statements, caring for patients with COVID-19 also had some positive outcomes for caregivers: promoting spirituality, improving relationships, and growth.

#### Promoting spirituality

Most participants mentioned that caring for patients with COVID-19 showed them that life could be shorter than they thought. Thus, they realized that this world and its material gains were not worth the attachment. They concluded that human beings should not immerse themselves in materialism and luxury. Most of the participants also believed that this period strengthened their faith in God. In other words, they felt that their relationship with God got stronger and their appreciation increased for the blessings of life, especially the blessing of health. In this regard, one of the caregivers said, *“My faith in God has peaked during this period because I heard that some people, physically healthier than my husband, died due to COVID-19 but my husband survived. This was mere grace of God.”* (Caregiver 7, caring for a husband, 37 years old).

#### Improving relationships

Most participants reported that caring for a patient with COVID-19 improved their relationship with the patient. As they noted the experience of living with and caring for someone for 2 weeks increased their interest in each other, and they appreciated each other during this period. Some caregivers stated that this experience increased their ability to forgive others, increased their tolerance threshold regarding the others' discouraging behaviors, and lowered their expectations of others, which improved their relationships with others.

“Now, I realized how important we are to each other, I feel we got closer.” (Caregiver 7, caring for a husband, 37 years old).

#### Growth

Some caregivers, especially younger individuals, stated that the experience of caring for a critically ill patient made them stronger and more responsible and provided them with a sense of growth.

“I became much stronger, as I gained experience and learned how to protect myself from fear and stress.” (Caregiver 9, caring for siblings, 27 years old).

## Discussion

This qualitative study explored how family caregivers experience and perceive caring for patients with COVID-19. Participants’ experiences in the caregiving period cause them to have some perceptions about their roles, responsibilities, and challenges they faced. Five main obtained themes of this study included “nature of the disease”, “unmet needs”, “unpleasant physical, psychological and social experiences”, “care facilitators,” and “positive experiences” reflect experiences of family caregivers of patients with COVID-19.

According to the findings of the study, family caregivers described the nature of COVID-19 as unknown because of fluctuating symptoms and unpredictability of the disease. There is limited information regarding the caring challenges of family caregivers in patients with COVID-19, but evidence shows formal caregivers, patients or the public community members experienced a wide range of concerns about the nature of COVID-19. For example, Sun et al. stated nurses participating in the study believe that COVID-19 is a new disease with an unpredictable condition that made them worry about secondary actions required if the patient's condition deteriorates [33]. Also, the results of a qualitative study on patients with COVID-19 indicated the disease appeared to manifest differently among the patients. The patients reported they had experienced symptoms of COVID-19 that sometimes got better and sometimes worse during different days of illness [34]. The result of studies revealed that unpredictability and uncontrollability of stressors are related to depression and anxiety [23, 35]. We suggest conducting quantitative studies to survey the effect of caring for patients with COVID-19 on the psychological status of caregivers.

In the current study, most participants had needs that were not met, including lack of knowledge, lack of health facilities, and financial problems. Lack of public knowledge and awareness regarding various aspects of coronavirus has been reported in previous studies [33, 36]. To reduce mortality and morbidity caused by COVID-19, accurate and efficient information should be provided to the general population, especially high-risk groups, by and healthcare workers and social media.

With the onset of the outbreak of COVID-19, the lack of medical facilities, equipment, and staff has affected health care systems and community members around the world. The result of some studies showed that lack of access to medical equipment for caregivers made caring for COVID-19 patients a challenge [23, 37]. Also, the high cost of COVID-19 treatment has affected families’ financial stress and well-being, where the immediate and long-term effects of COVID-19 have led to occupational vulnerability of families because of unemployment, declining incomes, debt, and economic hardship [21, 38].

Most of the respondents had experienced unpleasant physical, psychological, or social experiences while caregiving. The result of a review showed that sleep disorders, fatigue, and inadequate self-care were recognized as common physical health problems in family caregivers [39]. Family caregivers may also grapple with anxiety, depression, apathy, disappointment, loneliness, and isolation because of reduced social interactions and social exclusion while providing that care [16, 40]. All of these experiences may result in a poor quality of life among them. Therefore, implementing supportive interventions for family caregivers is essential if the health care system aimed to reduce the cost of care through home caregiving. These interventions can include easy access to healthcare facilities and medical consultation anytime, financial assistance, respite care, mental health counseling as well as a family caregiver education and training program to respond effectively to disease-related problems.

Along with the experienced challenges and problems, our participants reported some personal facilitators, including social support, adaptive mechanisms, and intrinsic motivations that help them to provide care for COVID-19 patients. Research findings have well established the relationship between social support and increasing the quality of caregiving. In the presence of social support, patient care becomes a valuable experience for family caregivers in addition to meeting patient needs. Also, emotional satisfaction, positive well-being, better relationships and stronger social bounds are more reported in caregivers who have received adequate social support from family members or others [9, 41]. Moreover, consistent with our findings, various adaptive strategies have been employed to deal with home caregiving in other studies. Looking for distractions, resting, discussing experiences and emotions [42], talking to a spiritual person, attending religious services, and praying and meditating are some coping mechanisms that have been reported in the literature [43]. Abendroth et al. also argued that the anxiety about caring for a patient at home can be reduced by seeking information in caregivers as an adaptive strategy [44]. In this study, participants stated a sense of responsibility and interest regarding the care of the patient. Similar to this finding, Zahed et al., reported that family caregivers considered caregiving as an honorable duty or a commitment to the love and care they shared as a spouse [45].

Due to increased number of COVID-19 cases and resource constraints in health care systems, the role and responsibilities of the family caregiver has become more important. In such pandemic emergencies, health policymakers and other community members must provide a comprehensive system of support for them. A system that, in addition to provide informational and instrumental support such as up-to-date information about disease and care, flexible working hours schedule and cash allowance for care, encourages them to continue their care. Health care staffs need to be clear about what family caregivers can do to help patients and also work to strengthen the sense of self-efficacy and empowerment in this crisis. Due to limited physical contact, a telehealth support service should be developed for family caregivers' education and consultation. Using telephone*-*based follow-up and web-based technology with features such as educational posters and videos and online chat sessions may be effective strategies to facilitate efficient and effective cares.

Emotional support from community members can also be strengthened with more empathy for patients and those affected by COVID-19 to reduce their loneliness and social isolation. Faith leaders and religious institutions are other good resources of support. They can perform online religious activities to reduce caregivers stress and also they can create online support groups for exchange of spiritual experiences. Finally, researchers need to do more study to have a complete picture of the needs, shortcomings and challenges of caregiving at home. 

### Limitations and strengths

In this study data saturation reached after interviewing with 12 female and only one male caregiver. Other experiences might have been described if more male family caregivers had been interviewed. For this reason and because of the nature of qualitative research, the results may not be generalizable to other family caregivers. However, data are gathered from interviews with family caregivers from both rural and urban areas and different age groups. The strength of this study is that it provided the family caregivers' perceptions of caring for COVID-19 patients, which were not studied previously. It seems a qualitative approach could be an appropriate method for studying experiences of family caregivers.

## Conclusion

According to the results of the study, paying more attention to the problems and needs of home caregivers of COVID-19 patients is of paramount importance. The health care system should provide adequate information and financial support to patients' families. Also, the psychological atmosphere of the society should be friendly to patients and their families to create more empathy and facilitate the care of patients at home. Emphasis on positive experiences can also provide more satisfaction to caregivers to continue caring.

## Supplementary Information

**Additional file 1.** Interview guide (translated from Farsi).

## Data Availability

All data analyzed during this study are included in this published article and its additional files.
