# Supplementary material for: Experiences of family caregivers of patients with COVID-19
Source: BMC Fam Pract. 2021 Jun 29;22:137. doi: 10.1186/s12875-021-01489-7 (PMC8241402; doi:10.1186/s12875-021-01489-7)
Supplement: Supplementary file 1 — Additional file 1. Interview guide (translated from Farsi). [file 12875_2021_1489_MOESM1_ESM.docx]

**[Family Caregivers' Experiences of Caring for Patients with COVID-19: A Phenomenological Study](https://pubmed.ncbi.nlm.nih.gov/25967646)**

Tahereh Rahimi, Neda Dastyar, Foozieh Rafati

**راهنمای مصاحبه**

**الف) سوالات اصلی**

1. لطفا تجارب مراقبت از بیمار مبتلا به کرونا ویروس را شرح دهید.
2. چه تجارب منفی در هنگام مراقبت از بیمار مبتلا به کرونا ویروس داشتید؟
3. چه تجارب مثبتی در هنگام مراقبت از بیمار مبتلا به کرونا ویروس داشتید؟
4. لطفا مشکلات و چالش هایی که در مراقبت از بیمار مبتلا به کرونا ویروس داشتید بیان کنید.
5. لطفا تسهیل کننده هایی که به شما در انجام وظایف و مسئولیتهای مراقبتی کمک می کرد را بیان کنید.

**ب) سوالات پیگیری**

1. می توانید بیشتر توضیح دهید؟ (در هر سوال در صورت نیاز)

2. می توانید یک مثال بزنید؟

**ج) در پایان مصاحبه**

آیا چیز دیگری هست که بخواهید اضافه کنید و از شما نپرسیده باشم؟
